# Secondary endoscopic ultrasound-guided gastroenterostomy to solve stent dysfunction and prolong lifetime in malignant gastric outlet obstruction

**DOI:** 10.1055/a-2643-8483

**Published:** 2025-08-08

**Authors:** Xintong Zhang, Lei Wang, Shanshan Shen

**Affiliations:** 1 66506 Department of Gastroenterology, Nanjing Drum Tower Hospital, Affiliated Hospital of Medical School, Nanjing University, Nanjing, China

Endoscopic ultrasound-guided gastroenterostomy (EUS-GE) is effective and safe to relieve gastric outlet obstruction (GOO) 1 . Long-term stent dysfunction, including stent occlusion, migration, and delamination, could be resolved by food disimpaction, stent exchanges, or an overlapping stent 2 3 4 . Here, we report a patient with pancreatic ductal adenocarcinoma (PDAC) who received secondary EUS-GE to solve stent dysfunction 10 months after the index operation.

A 54-year-old male with a history of uncinate process PDAC was admitted due to the recurrence of abdominal distention and vomiting 292 days after his first EUS-GE. The anastomotic lumen-apposing metal stent had maintained patency for 10 months, providing substantial nutrition for anti-tumor therapy and enabling a prolonged survival. This time, abdominal computed tomography demonstrated the recurrence of GOO ( Fig. 1 ). Gastroscopy revealed pills accumulated in the stomach, and erosion, necrosis and stenosis of intestine around the stent ( Fig. 2 ). The distal end of the stent was pointed to the afferent limb. The stent-efferent limb angle was so sharp that the endoscope could hardly pass through. Gastroscopy revealed multiple ulcers in the efferent limb. Radiography showed segmental narrowing of the intestinal lumen ( Fig. 3 ). We postulated this could be attributed to localized inflammatory edema. As the original pathway was passable but not smooth, we applied secondary free-hand EUS-GE elsewhere instead of stent repatency or replacement. A nasobiliary catheter was placed to instill saline solution and methylene blue, facilitating direct puncture with 15-mm × 10-mm cautery-enhanced LAMS (Hot Axios stent; Boston Scientific, USA) under EUS (GF-UCT260; Olympus, Tokyo, Japan) guidance from the upper part of gastric body's posterior wall ( Video 1 , Fig. 4 ). The patient tolerated a liquid diet from postoperative day 3 and gradually resumed a semiliquid diet.

**Fig. 1:**
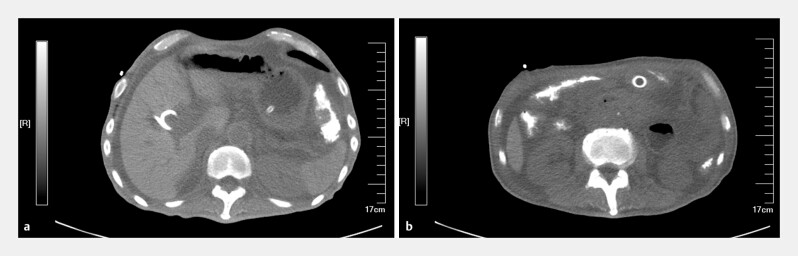
Postoperative abdominal computed tomography. **a** Stomach distention with accumulation of gastric contents. **b** Initial EUS-GE with lumen-apposing metal stent.

**Fig. 2:**
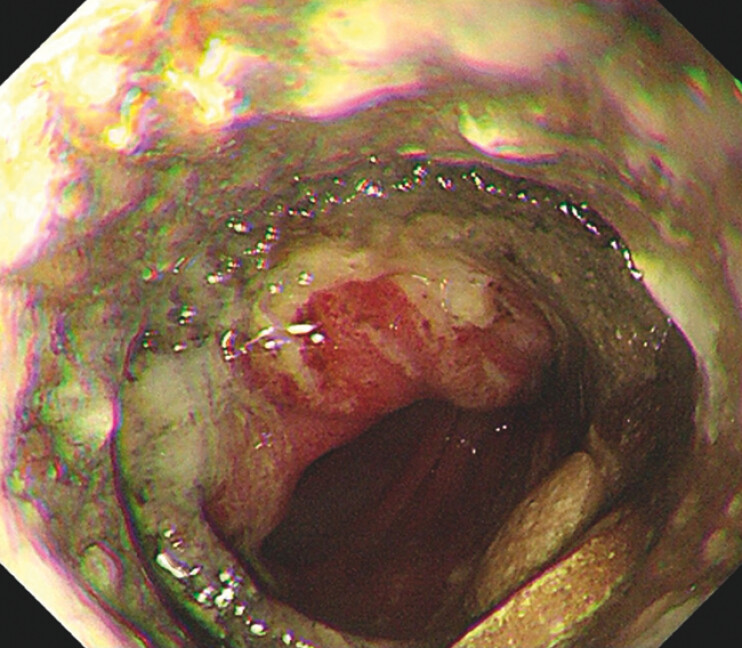
Endoscopy revealed erosion, necrosis, and stenosis of intestine around the stent.

**Fig. 3:**
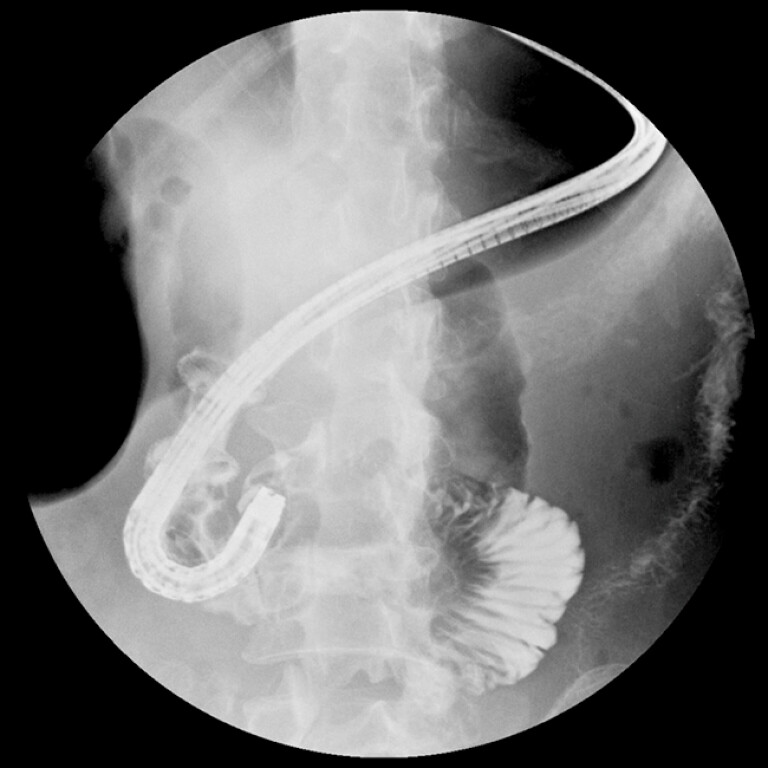
Radiography showed segmental narrowing of the efferent intestinal lumen.

**Fig. 4:**
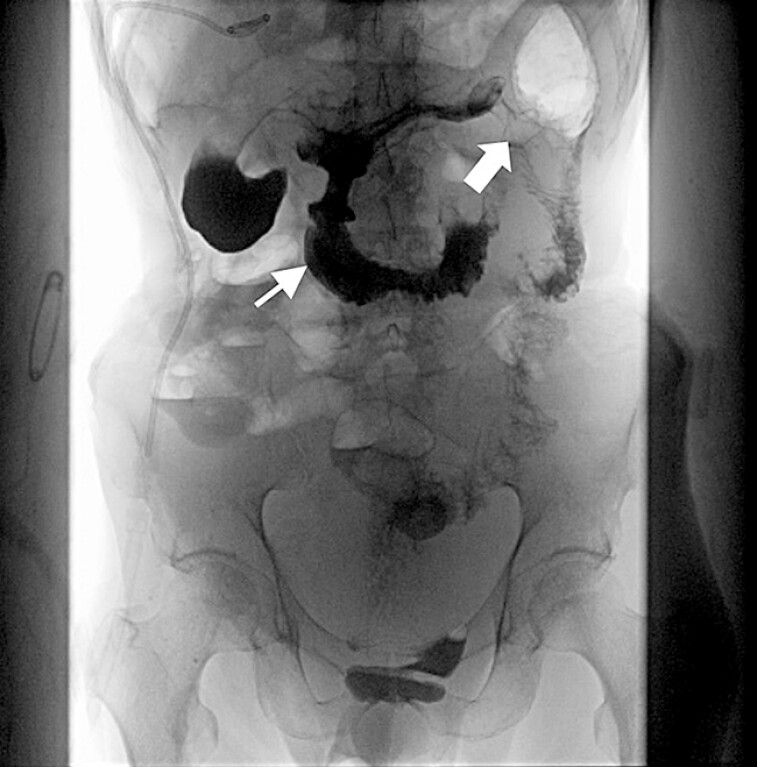
Postoperative radiography demonstrated stent patency after the secondary EUS-GE (thin arrow: contrast agent passed the initial stent but limited to the afferent limb; bold arrow: contrast agent passed the second stent and reached the distal jejunum).

Procedure of two EUS-GE for the patient.Video 1

We demonstrate the significance of EUS-GE to prolong survival duration in malignancy. We also provide a feasible resolution to prevent stent dysfunction for stent-dependent patients.

Endoscopy_UCTN_Code_TTT_1AS_2AK

## Notes

Endoscopy E-Videos https://eref.thieme.de/e-videos
*E-Videos* is an open access online section of the journal *Endoscopy* , reporting on interesting cases and new techniques in gastroenterological endoscopy. All papers include a high-quality video and are published with a Creative Commons CC-BY license. Endoscopy E-Videos qualify for HINARI discounts and waivers and eligibility is automatically checked during the submission process. We grant 100% waivers to articles whose corresponding authors are based in Group A countries and 50% waivers to those who are based in Group B countries as classified by Research4Life (see: https://www.research4life.org/access/eligibility/ ). This section has its own submission website at https://mc.manuscriptcentral.com/e-videos .
